# *In vivo* therapeutic effects of colorectal cancer cell-derived exosomes

**DOI:** 10.22038/ijbms.2020.46465.10730

**Published:** 2020-11

**Authors:** Ali Ganji, Iman Farahani, Mana Shojapour, Ali Ghazavi, Ghasem Mosayebi

**Affiliations:** 1 Molecular and Medicine Research Center, Arak University of Medical Sciences, Arak, Iran; 2 Department of Microbiology and Immunology, School of Medicine, Arak University of Medical Sciences, Arak, Iran; 3 Traditional and Complementary Medicine Research Center (TCMRC), Arak University of Medical Sciences, Arak, Iran

**Keywords:** CT26 colorectal cancer, Cytotoxic T-Lymphocyte, Exosome, Interferon-gamma, Tumor-derived exosomes

## Abstract

**Objective(s):**

Exosomes are nano-sized structures with lipid bilayer membranes that can be secreted by cancer cells. They play an important role in the biology of the tumor extracellular matrix. Exosomes may contain and transfer tumor antigens to dendritic cells to trigger T cell-mediated anti-tumor immune responses.

**Materials and Methods:**

BALB/c mice bearing CT26 colorectal cancer were treated subcutaneously with purified exosomes from analogous tumor cells. The mice were analyzed with respect to tumor size, survival, and anti-tumor immunity responses, including gene expression of cytokines and flowcytometry analysis of T lymphocytes.

**Results:**

The rate of tumor size growth in the exosome-treated group significantly decreased (*P*<0.05), and the flow cytometry results showed a significant reduction in the spleen regulatory T cells (Tregs) count of the exosome-treated group, compared with the untreated group (*P*=0.02). Although the increase in the serum level of interferon-γ (IFN-γ) and the number of cytotoxic CD8 T lymphocytes (CTLs) in the spleen tissue was not significant (*P*>0.05), the gene expression of IFN-γ increased significantly (*P*=0.006).

**Conclusion:**

The present results revealed that subcutaneous administration of tumor-derived exosomes could effectively lead to the inhibition of tumor progression by decreasing the number of Treg cells and up-regulation of the IFN-γ gene. Therefore, tumor-derived exosomes can be used as potential vaccines in cancer immunotherapy.

## Introduction

Cancer remains one of the leading causes of mortality worldwide (1). There are different treatments for cancer, including surgery, chemotherapy, radiotherapy, and immunotherapy. Despite decades of rigorous studies on cancer treatment, application of the mentioned strategies can be problematic due to some obstacles, such as negative cytotoxic effects of chemotherapy agents on the adjacent normal tissues (2, 3). Moreover, cancer cells can evade immunological elimination by secretion of immunosuppressive cytokines. These findings have led to the development of immune-activating therapies with vaccines to induce antigen-specific immune responses (4). 

Recent evidence shows that tissue-specific proteins can serve as targets for cancer vaccines (5). The search for more specific cancer biomarkers is a novel and potential approach, considering the high specificity and capacity of these biomarkers to carry specific antigens, known as exosomes, which can trigger immune responses (4, 6). Exosomes, as nano-sized lipid bilayer membranes, are secreted by a variety of stromal and cancer cells into the extracellular matrix and can bind to or fuse with other cells through cell-to-cell contact (7, 8). Depending on the cell of origin, exosomes differ in quantity, features, and function. For example, exosomes from stromal cells involve proteins, messenger RNA (mRNA), and microRNA (miRNA) molecules (9, 10). It is known that cancer cells release more exosomes than normal cells (11). Exosomes are capable of delivering various tumor antigens, which can be used as potential carriers in cancer immunotherapy. They are stable in biological fluids, highly specific, and adherent to cells. They can also easily evade lung clearance and pass through the blood-brain barrier (11, 12).

According to several studies, exosomes are released from tumor cells and contain major histocompatibility complex (MHC) class I molecules and tumor antigens (13). In addition, exosomes, which outflow from malignant tumor cells (tumor-derived exosomes), carry tumor antigens and antigen-presenting molecules, triggering anti-tumor T-cell responses (14). Some studies have widely investigated the therapeutic application of exosomes for tumor therapy. In 2015, a study reported that migration of tumor-derived exosomes and dendritic cell (DC)-derived exosomes to the lymph nodes could activate CD4^+^ and CD8^+^ T cells, leading to the stimulation of anti-tumor responses (15). Although some promising studies and clinical trials have revealed the therapeutic effects of exosomes, there are still some uncertainties about the efficacy of exosomes for cancer therapy. In this regard, studies on the efficacy of cancer vaccines for lung cancer treatment show that they are effective for patients with non-small-cell lung carcinoma (NSCLC) (16). 

There are two major problems in cancer immunotherapy: limited access to a variety of cancer-associated antigens and lack of suitable delivery molecules for the transfer of these antigens to DCs. Tumor-derived exosomes are not only loaded with antigens of their originating cancer cells but are also efficient cargos for delivering these antigens to DCs (17). Tumor cell-derived exosomes can trigger immune responses in the body by carrying and delivering specific cancer antigens including HSP70, Her2/Neu, Mart1, TRP, and gp100 (13, 14). Due to the unique properties of exosomes, these particles have become promising candidates in cancer immunotherapy (18). In this respect, tumor derived-exosomes were evaluated in tumor transplant studies in mice. The main finding was that the transferred exosome from breast and colon cancer cells prevented tumor growth by stimulating CD4 and CD8 dependent T cells (13, 17). 

Generally, tumor-derived exosomes are critical mediators in the tumor microenvironment, organizing major events for tumor growth, metastasis, and development. To date, no study has evaluated immune system responses, such as T regulatory lymphocytes (Treg) and cytotoxic T lymphocytes (CTL) with their cytokines, or evaluated the subcutaneous injection of tumor-derived exosomes in tumor-bearing mice. Since exosomes can activate immune responses, they are potential targets for cancer treatment (19). Therefore, the aim of this *in vivo* study was to explore the efficacy of exosomes derived from colorectal cancer cells as vehicles for tumor cell antigens in cancer therapy. 

## Materials and Methods

***Cell culture***

Mouse colorectal cancer cell line, CT26, was purchased from the Pasteur Institute (Iran). The cells were cultured at 37 °C in a 5% CO_2_ atmosphere with 95% humidity in Dulbecco’s Modified Eagle’s Medium (DMEM) (Invitrogen, UK), supplemented with 2 mM L-glutamine (Gibco, USA) and 10% fetal bovine serum (FBS; Gibco, USA); the medium was changed every 48 hr.

***Exosome isolation and characterization***

After the CT26 cell culture reached 85–90% confluency, the cells were washed three times with phosphate-buffered saline (PBS) to remove the residual FBS-supplemented culture medium thoroughly and then resuspended in conical tubes, containing DMEM (FBS-free medium) for 24 to 48 hr at 37 °C. After incubation, 250 ml of the cell culture supernatant was centrifuged at 300×g for 10 min to remove the cells. Next, the supernatant was centrifuged at 16,000×g for 30 min to remove the debris and cells from the supernatant. Afterward, the cell-free supernatant was filtered using a 0.22 μm filter (Millipore, USA) to purify particles larger than 200 nm. Exosomes were isolated and purified using an exosome purification kit according to the manufacturer’s instructions (Exo-spin™, Cell Guidance Systems) (20, 21). The purified exosomes were characterized by size and shape, using transmission electron microscopy (TEM; LEO906, Germany). The collected exosomes (50 µl) were unloaded on a carbon-coated grid and fixed in 1% acetate (Merck, Germany). The samples were analyzed by TEM at 80 kV (20). 

***Mice, tumor challenge, and treatment***

Inbred female BALB/c mice (4–6 weeks old) were purchased from the Pasteur Institute (Iran). First, the mice were weighed and then housed under standard controlled conditions. Seven days after purchasing the mice, they were anesthetized on day zero, and then, 1×10^5 ^CT26 cells were inoculated in 100 µl of PBS and injected subcutaneously in the right flank of the mice. Every four days, the mice were weighed and examined for monitoring locally palpable tumors. After 10–14 days of tumor challenge, the mice without any palpable tumor sites were excluded from the study. Tumor-bearing mice were randomly divided into two groups (eight rats per group). The treatment phase started by injecting 50 µl (10 mg/ml) of exosome (exosome-treated) and 50 µl of PBS (untreated) subcutaneously one day after tumor challenge on days 1, 7, and 14.

***Tumor size measurement***

As soon as the tumor became palpable, its size (length and width) was measured every other day, using calipers by one person to avoid observational errors with an accuracy of 0.01 mm. For more accuracy, each measurement was repeated three times. The tumor volume was calculated based on the following formula:

 (length× (width)^ 2^)×0.52.

***Cytokine assay***

The levels of interferon-γ (IFN-γ) (sensitivity: 0.99 pg/ml) and transforming growth factor-β (TGF-β) (sensitivity: 8.6 pg/ml) were determined by analyzing the blood serum of mice via cardiac puncture under deep anesthesia with ketamine and xylazine on the sacrifice day, using ELISA kits according to the manufacturer’s guidelines (Thermo Fisher Scientific, USA). Each sample was tested in triplicate and quantified using a microplate reader (Stat Fax 2100, USA) at a wavelength of 450 nm. 

***Flow cytometry***

Twenty-five days after the tumor challenge, tumor-infiltrating lymphocytes were harvested from the euthanized mice on the sacrifice day. For this purpose, the skin was disinfected, and tumor tissues were carefully dissected. After washing the tissue with PBS, it was dissected into smaller fragments using a scalpel. Next, 5 ml of RPMI-1640 medium (supplemented with collagenase I, collagenase IV, DNase, and 10% FBS; Gibco, USA) was added, and the tumor was disintegrated by rubbing against the mesh. The cells were then filtered and treated with ammonium-chloride-potassium (ACK) lysing buffer (Quality Biological, USA) before cellular staining. For the evaluation of cytotoxic CD8 T lymphocytes (CTL), the suspended cells were incubated for 60 min at 4 °C with the FITC-conjugated anti-mouse CD3 antibody and PerCP-conjugated anti-mouse CD8 antibody. For Treg cell evaluation, the suspended cells were incubated for 60 min at 4 °C with PerCP-conjugated anti-mouse CD25, phycoerythrin (PE)-conjugated anti-CD4, and FITC-conjugated anti-mouse FoxP3. All antibodies were purchased from eBioscience (eBioscience, USA). Finally, each sample (10,000 events) was assessed using a three-color FACSCalibur flow cytometer (BD Biosciences, CA, USA), and the results were analyzed using the FlowJo software (Tree Star, Inc., OR, USA).

***Gene expression analysis***

For this purpose, total RNA was extracted from the tumor tissue using an RNA isolation kit (Favorgen, Taiwan), and cDNA was synthesized according to the manufacturer’s instructions (Fermentas, USA). Primers were designed using the AlleleID software (Primer Biosoft International, CA), and their specificity was examined by Primer-BLAST in the NCBI database (Table 1). Afterward, real-time polymerase chain reaction (PCR) was carried out in triplicate, using SYBR Green PCR Master Mix Kit (Fermentas, USA) in a LightCycler system (Roche, Germany). GAPDH gene was considered as the housekeeping gene. Non-specific bands and primer-dimers were determined via melting curve analysis. The Paffle method was applied, and the ratios were considered as the final results of statistical analysis.

***Statistical analysis***

Statistical analyses were performed using SPSS Version 16.0 (SPSS, Inc., Chicago, IL, USA). The assumption of normality was tested using the Kolmogorov–Smirnov test. Independent sample t-test was performed for the comparison of normally distributed variables. Data are presented as Mean±SEM. *P*-value less than 0.05 was considered statistically significant.

**Table 1.** PCR primer sequences for housekiping and cytokine genes

**Primers **	**Length**	**Sequences, 5'→ 3'**
**GAPDH**	224	
**Forward**		CGGTGTGAACGGATTTGG
**Reverse**		CTCGCTCCTGGAAGATGG
**IFN-γ**	200	
**Forward **		GAACTGGCAAAAGGATGGTGAC
**Reverse **		TGACCTCAAACTTGGCAATACTC
**TGF-β**	193	
**Forward **		AATTCCTGGCGTTACCTTGG
**Reverse **		GGCTGATCCCGTTGATTTCC

GAPDH: glyceraldehyde-3-phosphate dehydrogenase; IFN-γ: interferon-γ; TGF-β: transforming growth factor-β

**Figure 1:**
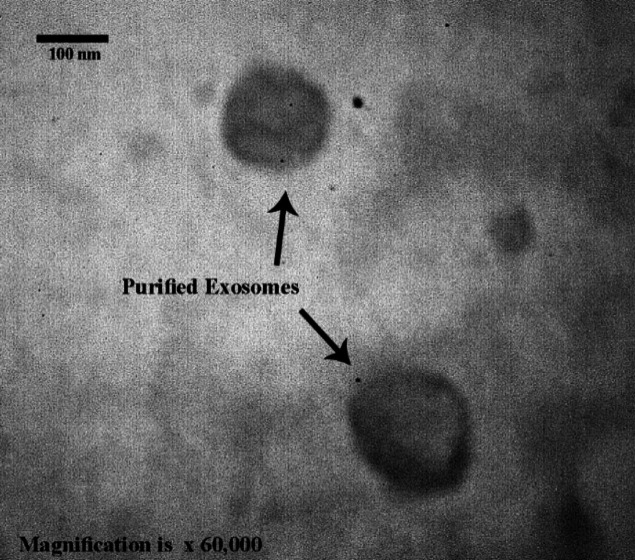
Imaging of exosomes by TEM at 80 kV (×60,000)

TEM: transmission electron microscopy

**Figure 2:**
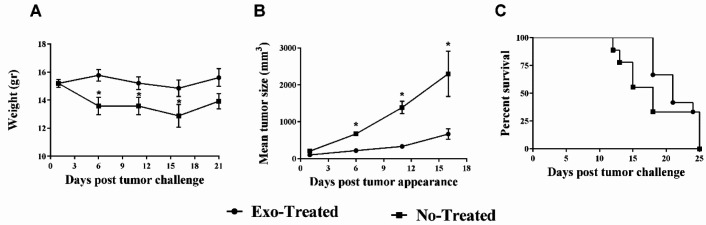
The animals’ weight, tumor size, and survival: (A) Weight assessment for 21 days after tumor challenge; (B) tumor volume measurements over 17 days after tumor appearance; and (C) survival analysis (*P<0.05)

**Figure 3:**
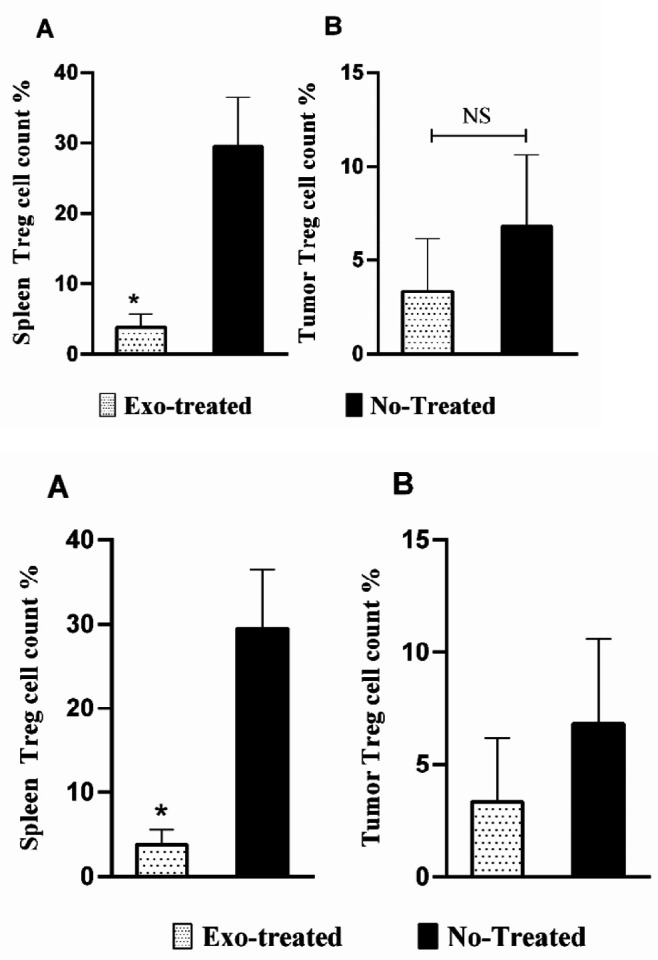
Cell count percentage of regulatory T lymphocytes in (A) the spleen tissue and (B) tumor tissue of the studied groups (*: *P*<0.05; NS: not significant)

**Figure 4.**
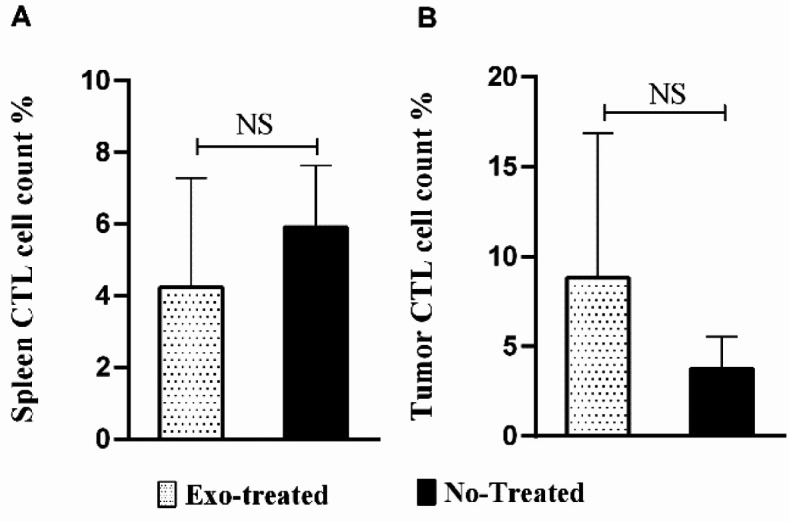
Cell count percentage of CTL in (A) the spleen tissue and (B) tumor tissue of the studied groups (NS: not significant)

**Figure 5:**
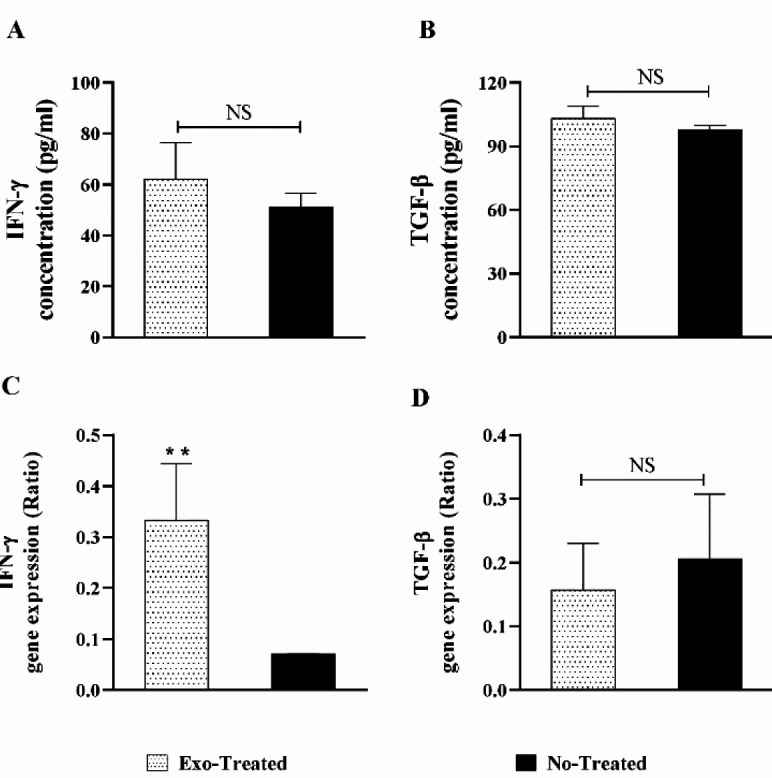
Evaluation of the serum concentrations of (A) IFN-γ and (B) TGF-β and gene expression of (C) IFN-γ and (D) TGF-β in the studied groups (**:*P*<0.01; NS: not significant)

## Results

***Exosome identification***

The purified exosomes were characterized regarding size and shape using TEM. The exosomes were round with a diameter range of 40–150 nm (Figure 1). 

***Evaluation of animals’ weight, tumor size, and survival***

The first day after the tumor challenge, the mean weight of the animals was the same in all three groups (15.2±0.28 g). The average weight of mice in the untreated group decreased significantly 6, 11, and 16 days after tumor inoculation in comparison with the exosome-treated group (*P*<0.05) (Figure 2A). The rate of tumor size growth in the exosome-treated group decreased significantly 6, 11, and 16 days after tumor inoculation, compared with the untreated group (*P*<0.05) (Figure 2B). The study of survival showed an insignificantly longer life span in the exosome-treated group in comparison with the untreated group (log-rank test; *P*>0.05) (Figure 2C).

***Treg cell count***

According to the results of flow cytometry, the number of spleen Treg cells in the exosome-treated group decreased significantly compared with the untreated group (*P*=0.02) (Figure 3A). Also, there was a meaningless reduction in the Treg cell count in the tumor tissues of the studied groups (*P*>0.05) (Figure 3B). 

***CTL cell count***

Flow cytometry results showed insignificant changes in the CTL of the spleen between the exosome-treated and untreated groups (Figure 4A). Moreover, there was an inconsiderable increase in tumor-infiltrated CTLs in the exosome-treated group, compared with the untreated group (*P*>0.05) (Figure 4B).

***Serum concentrations and gene expression of IFN-γ and TGF-β***

Evaluation of IFN-γ showed an insignificant increase in the exosome-treated group in comparison with the untreated group (*P*>0.05) (Figure 5A). The serum concentration of TGF-β showed an unimportant rise in the exosome-treated group in comparison with the untreated group (*P*>0.05) (Figure 5B). Analysis of gene expression showed significant up-regulation of IFN-γ gene expression in the exosome-treated group, compared with the untreated group (*P*=0.006) (Figure 5C). The difference in *TGF-β* gene expression between the groups was not significant (Figure 5D).

## Discussion

Exosomes are nanoparticles, which may originate from both normal and tumor cells and play an important role in the biology of the extracellular matrix (22, 23). The cells from which exosomes are derived determine their different and sometimes contradictory functions (24). In this study, the effects of exosomes derived from CT26, which contain diverse endogenous tumor antigenic peptides (25), were studied in a tumor-bearing mouse model. The main objective of this study was to investigate whether subcutaneously injected CT26 tumor cell-derived exosomes could exert any anti-tumor effects through immunomodulation of the immune system.

In the present study, there was a significant reduction in the tumor size of mice treated with tumor-derived exosomes after the intervention, compared with the control group without exosomal treatment. Also, the mean weight of mice treated with exosomes was significantly higher than that of the control group. In this regard, Zhang *et al.* showed that the derived exosomes from human embryonic kidney (HEK) 293T cells, as carriers of hepatocyte growth factor (HGF) siRNA, could inhibit the HGF-c/MET pathway and prevent tumor growth and angiogenesis in gastric cancer; the decreased tumor size in their study is in agreement with the findings of our study (18).

Different studies have indicated more efficient anti-tumor responses of T cells by using exosomes from pulsed DCs with tumor peptides. According to the previous study, tumor peptide-pulsed DC exosomes could activate CTL, inhibit tumor cell growth, and significantly reduce tumor size in the studied mice (19). Furthermore, Wolfers *et al.* investigated the therapeutic properties of murine tumor-derived exosomes, which were inoculated with DCs *in vitro*. Pulsed DCs with tumor cell-derived exosomes were injected into mice, resulting in a significant delay in the tumor growth of treated mice, compared with the untreated group (13). The results of this study are consistent with our findings and the only difference was that in the study of Wolfers *et al*. the exosomes were exposed to DC *in vitro*, while in our study, tumor exosomes containing tumor-specific antigens were injected subcutaneously for uptake by dermal DCs. Although numerous studies on tumor-derived exosomes have shown that these exosomes contribute to tumor growth, it is speculated that subcutaneous injection of tumor-derived exosomes can promote the efficient uptake of exosomes by dermal DCs. It can also lead to the stimulation of cancer antigen-specific T lymphocytes via MHC complexes and co-stimulatory molecules (26, 27). Subcutaneous injection of antigens is the most efficient way to induce immunogenicity; therefore, many types of vaccines are preferred to be administered subcutaneously (28).

IFN-γ gene expression increased significantly in the treated group, compared with the control group. This may indicate that T helper 1 (Th1) cells and natural killer (NK) cells have enhanced cellular immunity, as IFN-γ cytokine is mainly secreted by Th1 and NK cells. In this regard, research showed that macrophage-derived exosomes can stimulate DCs and result in the further production of IFN-γ by T cells and NK cells, which in turn enhances Th1 cell responses (29). The serum levels of TGF-β and IFN-γ decreased insignificantly in the exosome-treated group, compared with the untreated group; however, it is speculated that this difference might have been significant over a longer period.

In spite of the insignificant reduction in the number of Treg cells in the tumor tissue, the number of Treg cells in the spleen tissue decreased significantly. Treg cell reduction can modulate immune responses to induce anti-tumor immunity by activating T cells and NK cells; this may be one of the reasons for the reduction of tumor growth in the exosome-treated group (30). Histological results showed that the number of infiltrated CTLs in the tumor tissues of the treated group was significantly higher than that of the control group. The increasing number of CTLs in the exosome-treated group, compared with the untreated group, was also insignificant, although, over a longer period, the difference in CTL infiltration into the tumor tissue could be significant between the exosome-treated and untreated groups, since the results of this experiment are in line with the IHC results. Likewise, researchers suggested that tumor-derived exosomes induce tumor-specific immune responses in CTLs, resulting in anti-tumor immunity in animal models (17).

## Conclusion

In this study, subcutaneously injected CT26 tumor-derived exosomes in tumor-bearing BALB/c mice could successfully inhibit tumor progression and increase the infiltration of CTLs in the tumor tissue. Our results suggest that the significant decrease in the number of Treg cells and up-regulation of IFN-γ gene produced such anti-tumor responses. The beneficial properties of tumor-derived exosomes, including their function as suitable antigen cargos and bearing specific cancer antigens, have made these nano-sized particles a convenient vaccine candidate in cancer immunotherapy. However, the analysis of a larger population of tumor-bearing mice within a longer period is suggested in future studies.

## Funding

This study was funded by Arak University of Medical Sciences (grant number IR.ARAKMU.REC.1395.162).

## Research Involving Human participants and Animals

All procedures performed in this study involving animals were in accordance with the ethical standards of the Animal Ethics Committee of Arak University of Medical Sciences (IR.ARAKMU.REC.1395.162) and with the 1964 Helsinki declaration and its later amendments or comparable ethical standards.

## Conflicts of Interest

The authors declare no competing interest.
